# Detecting possible pairs of materials for composites using a material word co-occurrence network

**DOI:** 10.1371/journal.pone.0297361

**Published:** 2024-01-26

**Authors:** Chika Ishii, Kimitaka Asatani, Ichiro Sakata

**Affiliations:** 1 Customer Experience Department, Cisco Systems G.K., Minato-ku, Tokyo, Japan; 2 Department of Technology Management for Innovation, Graduate School of Engineering, The University of Tokyo, Bunkyo-ku, Tokyo, Japan; University of Sharjah, UNITED ARAB EMIRATES

## Abstract

Composite materials are popular because of their high performance capabilities, but new material development is time-consuming. To accelerate this process, researchers studying material informatics, an academic discipline combining computational science and material science, have developed less time-consuming approaches for predicting possible material combinations. However, these processes remain problematic because some materials are not suited for them. The limitations of specific candidates for new composites may cause potential new material pairs to be overlooked. To solve this problem, we developed a new method to predict possible composite material pairs by considering more materials than previous techniques. We predicted possible material pairs by conducting link predictions of material word co-occurrence networks while assuming that co-occurring material word pairs in scientific papers on composites were reported as composite materials. As a result, we succeeded in predicting the co-occurrence of material words with high specificity. Nodes tended to link to many other words, generating new links in the created co-occurrence material word network; notably, the number of material words co-occurring with graphene increased rapidly. This phenomenon confirmed that graphene is an attractive composite component. We expect our method to contribute to the accelerated development of new composite materials.

## Introduction

Interest in composites has increased recently, and the composite materials market is estimated to increase from USD 88.0 billion in 2021 to USD 126.3 billion by 2026 [1]. Composites are materials that consist of two or more composition materials with considerably different chemical or physical properties. Composite materials are popular because they have two or more properties. For instance, carbon fiber reinforced polymers (CFRPs), one of the most popular composites, have both strength and lightness. CFRPs are applied in various fields, such as automotive and aerospace engineering, to reduce costs and energy consumption [2]. Among composites of materials, those with completely different properties, such as Prussian blue and cellulose have attracted particular attention in recent years [3]. Morinobu Endo, one of the pioneers of carbon nanofibers and carbon nanotubes (CNTs), is working on the compounding of innovative material combinations, such as CNTs and polymer materials [4–6]. However, developing new composite materials is time-consuming, as one study reported that the development of new materials takes over 20 years [7]. One reason for this phenomenon is that there is a very large number of potential material options; MatWeb, an online material database, has data on over 170,000 materials [8]. Since many material combinations can be combined, finding new and compositable pairs of materials from them is difficult.

To solve this problem, material informatics (MI), which is an academic field combining material science and computer science, is attracting attention [9]. This academic discipline aims to accelerate the process of designing and finding new materials by experimental data analysis. Some studies have reported that data analysis methods, such as neural networks, similarity measurements, and data mining, can be applied to predict the physical characteristics of new composites [10–12]. Although MI is a new field, it has made rapid progress, leading to the development of high-performance composites [13].

However, some materials are unsuitable for MI; for example, some polymers have physical property values that are difficult to calculate and are unsuitable for MI [14]. The limitations of candidate materials in MI may lead to the overlooking of new combinations of materials. Thus, developing a method to investigate materials from a wider viewpoint to find new pairs is needed.

MI is notable; however, the usefulness of bibliometric networks for discovering new materials has been proven. One study succeeded in predicting new heat-conductive materials with a network of knowledge extracted from scientific publications [15]. Since new scientific knowledge is generated in existing knowledge networks, it is important to consider a prior knowledge network of material science to predict new materials [16]. Scientific papers contain various information about scientific knowledge relationships, such as citation relationships. Regarding journal papers on composite materials, the authors describe pairs of materials selected as components for composites; “Graphene-Polyaniline” and “TiO_2_/Graphene” are examples of material pairs [17, 18].

Link prediction in a network is a method to detect a possible combination between many candidates. This technique predicts the existence of a link between two nodes from structural changes in a network. Link prediction is applied in various situations, such as in the prediction of technological spinoffs that are used in unanticipated field technologies in an industry and in the combinations of promising research collaborators; link predictions of networks of interacting proteins have been applied to predict protein functions [19–21]. There are several link prediction methods, one of which is based on information related to the network structure [22]. This technique refers to information that describes the link structure around the nodes, for example, follower/followee relationships on social networks and protein‒protein interaction relationships. Examples of network structure indexes are the common neighbor (CN), Jaccard coefficient (JC), resource allocation index (RA), Adamic/Adar index (AA), and preferential attachment (PA) [23–27].

By considering the usability of bibliometric networks for discovering new materials and link prediction techniques for detecting possible pairs, we hypothesize that new composite materials can be predicted by performing link prediction on the co-occurrence networks, with the material words described in the paper being nodes and their co-occurrence relationships being links. The purpose of this study is to predict compositable pairs of materials from a larger number of candidates than in previous studies for investigating materials from a broader perspective to discover new combinations. We assume that using bibliographic information has the potential to consider a larger number of materials than physical information because bibliographic information contains data on a high number of materials; additionally, information on materials described in academic papers can be extracted from databases. More than 150000 papers on composite materials are stored in Web of Science (WoS) databases, which is an online subscription-based scientific citation indexing service maintained by Clarivate Analytics. In this study, we extract the bibliometric information of materials and conduct link prediction of the co-occurrence network of material words to detect new and compositable material pairs.

## Methods

### Method outline

**Fig 1:**
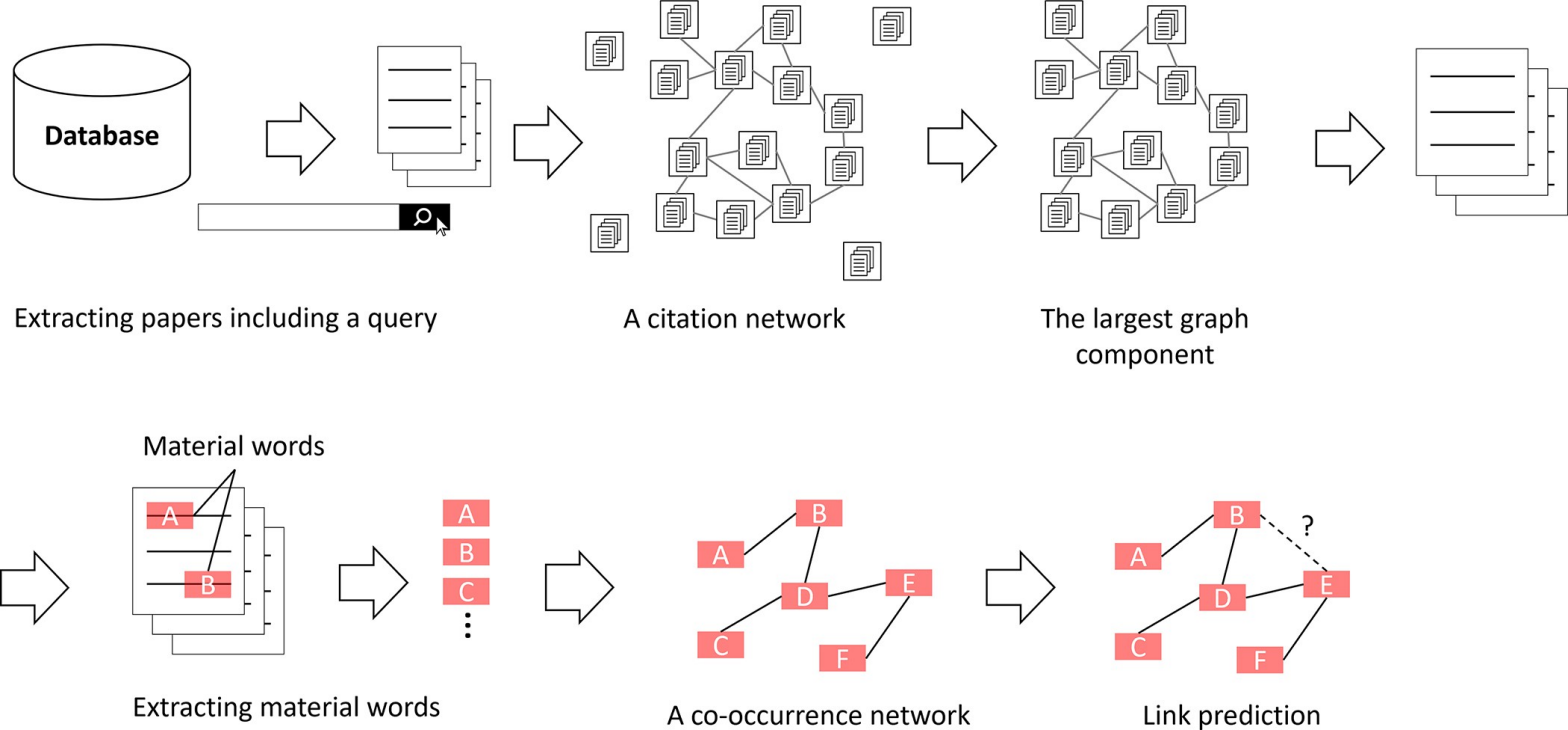
Method outline.

Our method involved the following four steps (Fig 1). The details of each step are as follows:

- Extracting papers on composite materials
- Listing material words from collected papers
- Creating a co-occurrence network of material words
- Link prediction of a co-occurrence network of material words

### Extracting scientific journal papers describing composite materials

To extract academic papers on composite materials, we obtained bibliographic data related to composite materials from the Science Citation Index and the Social Science Citation Index gathered by the Institute for Science Information. We used WoS for accessing these databases to collect academic articles published over a wide range of years because the WoS databases include journal publication records from a broader span of years than those of other databases.

We analyzed a citation network as follows. First, we searched for academic publications from the period before 8/15/2020 using the query “composite material*” (the asterisk* indicates a wildcard that can help to locate the appropriate results). Second, we created a citation network of the extracted papers with the obtained citation data. Third, papers not linked to others were excluded because we considered papers without citations of other studies to be irrelevant to the primary subject (composite materials). In this study, we analyzed the extracted papers included in a maximum component of a citation network.

### Listing material words from collected papers

We extracted material words from the collected academic articles and counted the numbers of scientific papers in which each material word occurred by following an academic landscape system [28]. We selected the 100 most frequently appearing material words and named them “the 100 material words”. We only analyzed material words with high frequencies of occurrence because material words with low frequencies were less likely to co-occur with others.

### Creating a co-occurrence network of extracted material words

We examined whether the two specified material words co-occurred or not, that is, whether there was a journal including both of them. We created a co-occurrence network of nodes and links representing the 100 material words and their co-occurrence relationships, respectively.

### Link prediction of a co-occurrence network

We defined three training periods (TRP1 (1/1/2010–12/31/2012), TRP2 (1/1/2011–12/31/2012), and TRP3 (1/1/2012–12/31/2012)) and one testing period (TSP (1/1/2013–2015/12/31)). Generally, the training/testing periods used for link prediction are 5–10 years; however, shorter periods were selected for this study [29, 30]. We concluded that shorter periods were appropriate for analyzing the changes in this research field because these changes have rapidly increased in recent years; the rate of increase in the number of scientific papers related to this topic has risen to a similar degree [31].

We created five co-occurrence networks using the extracted papers published on dates before 12/31/2009, 12/31/2010, 12/31/2011, 12/31/2012, and12/31/2012. Material word pairs that co-occurred for the first time in each period were identified by taking the difference in the material word co-occurrence network between the start and end of each period. For example, for TRP1, links with co-occurrence networks were not found in the extracted papers that were published by 12/31/2009, but links were found in the extracted papers that were published by 12/31/2012; therefore, that date was regarded as the first instance of co-occurrence for TRP1.

Next, we calculated the scores of the material word pairs with the network structure index. We used eight network structure indexes—CN, JC, RA, AA, PA, common neighbors using community information (CNSH), the internal resource allocation index using community information (RASH), and the intercluster measure (WIC)—because network structure indexes were deemed appropriate for the link prediction of small networks [32]. Each node pair (*x*, *y*) index was calculated according to the following equations (Table 1) [23–27, 33–35]. In this study, CNSH, RASH, and WIC were calculated with the material class; the details of this step are described in the results section as community information.

**Table 1:** Score for node pairs {*x*, *y*} under link prediction using each index (where Γ(*x*) = the number of neighbors of node x in the cooccurrence network, f(u) = 1 if x and y belong to the same community and f(u) = 0 otherwise, and *δ* = the arbitrary constant (the Default Value is 0.001)).

Network index	Equation	Network index	Equation
CN	Γ(x)∩Γ(y)	PA	|Γ(x)||Γ(y)|
JC	$\frac{\left|\Gamma (x)|\cap |\Gamma (y)\right|}{\left|\Gamma (x)|\cup |\Gamma (y)\right|}$	CNSH	$\Gamma (x)\cap \Gamma (y)+\sum_{u\in \Gamma (x)\cap \Gamma (y)}f(u)$
RA	$\sum_{u\in \Gamma (x)\cap \Gamma (y)}\frac{1}{\left|\Gamma (u)\right|}$	RASH	$\sum_{u\in \Gamma (x)\cap \Gamma (y)}\frac{f(u)}{\left|\Gamma (u)\right|}$
AA	$\sum_{u\in \Gamma (x)\cap \Gamma (y)}\frac{1}{\log\left|\Gamma (u)\right|}$	WIC	$\frac{\left|\Lambda_{x,y}^{W}\right|}{|\Lambda_{x,y}^{IC}|+\delta }$

**Fig 2:**
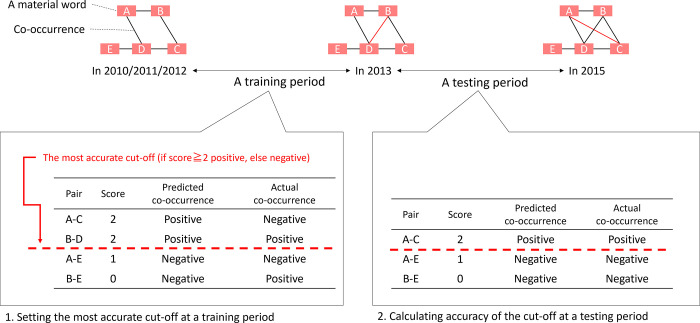
Link prediction outline.

**Fig 3:**
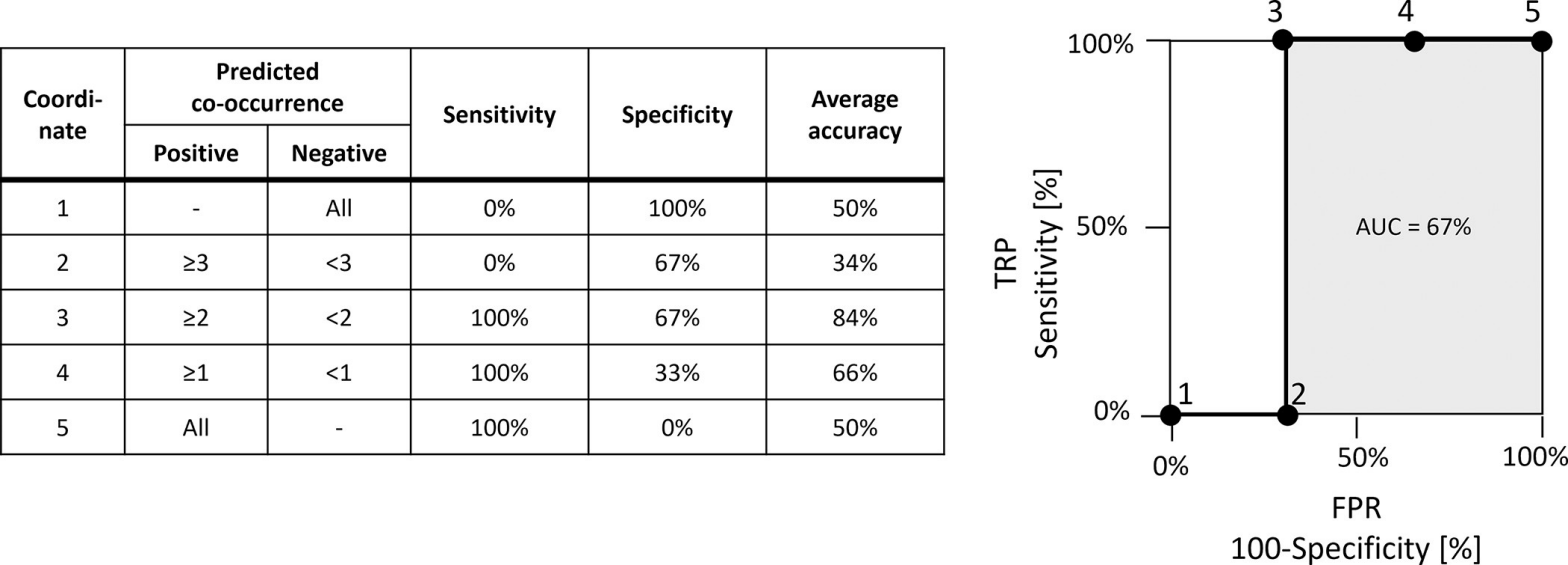
Example ROC curve and AUC graph.

Using the score of the network index, we judged which combinations of training periods and network indexes were the most appropriate for link prediction with the following steps. First, we counted the true positives (TPs), false positives (FPs), true negatives (TNs), and false negatives (FNs) of the link prediction process by setting each score as a cutoff in a training period; from there, we selected the best cutoff of each combination of a training period and a network index (Fig 2). TP and TN were outcomes where the link prediction correctly predicted the positive and negative classes, respectively, whereas FP and FN were outcomes where the link prediction incorrectly predicted the positive and negative classes, respectively. Second, we calculated the sensitivities and specificities from the TPs, FPs, TNs, and FNs with Formulas (1) and (2) [36]. The sensitivities and specificities represent the accuracies of the positive and negative predictions, respectively. In our study, sensitivity or specificity referred to the discovery accuracies of pairs of materials that could or could not be composited. Third, we plotted the receiver operating characteristic (ROC) curves, displaying (sensitivity, 1–specificity), as shown in Fig 3. Finally, we calculated the average accuracies and areas under the curve (AUCs) and evaluated the accuracies of the link prediction processes in each of the 24 cases (the cases were combinations of eight network structure indexes and three training periods) based on these values. The average accuracy was determined by the average values of the sensitivities and specificities (Formula (3)). AUC represented the area between the horizontal axis and the ROC curve, plotting (sensitivity, 1–specificity), as shown in Fig 3 and Formula (4) [37]. The AUC calculation required a value of area from 0% to 100%: the higher the area values were, the more accurate the link prediction [38].


$$Sensitivity=\frac{TP}{TP+FN}$$ (1)

$$Specificity=\frac{TN}{TN+FP}$$ (2)

$$Average\;accuracy=\frac{Sensitivity+Specificity}{2}$$ (3)

AUC=∫(Sensitivity)d(Specificity) (4)

## Results

### Extracting scientific journal papers on composite materials

A total of 75076 papers containing at least one keyword were collected. We focused on the maximum connected component, which accounted for approximately 59% of the total papers (44430 of 75076 papers).

### Listing material words from collected papers

The 100 material words extracted from the collected papers were classified into 4 classes: carbon, ceramic, metal and organic materials (Table 2); the numbers of material words categorized into each class were 7, 41, 10 and 42, respectively.

**Table 2:** The 100 material words in each material class (listed in descending order of occurrence number).

Material class “carbon”: CNT, graphite, graphene, diamond, boron carbide (B_4_C), fullerene, carbon nitride (C_3_N_4_)
Material class “ceramic”: silica (SiO_2_), silane (SiH_4_), boron nitride (BN), silsesquioxane, kaolinite, melamine, zeolite, polysiloxane, titanium dioxide (TiO_2_), alumina, aluminum oxide (Al_2_O_3_), zinc oxide (ZnO), calcium phosphate (Ca_3_(PO_4_)_2_), barium titanate (BaTiO_3_), zirconium dioxide (ZrO_2_), calcium carbonate (CaCO_3_), copper oxide (CuO), iron(III) oxide (Fe_2_O_3_), manganese oxide (MnO_2_), silver nitrate (AgNO_3_), nickel oxide (NiO), tin oxide (SnO_2_), cerium oxide (CeO_2_), iron(III) chloride (FeCl_3_), magnesium oxide (MgO), titanium diboride (TiB_2_), cobalt ferrite (CoFe_2_O_4_), molybdenum disulfide (MoS_2_), lithium iron phosphate (LiFePO_4_), tricobalt tetroxide (Co_3_O_4_), vanadium oxide (V_2_O_5_), lithium vanadium (Li_3_V_2_), calcium chloride (CaCl_2_), molybdenum trioxide (MoO_3_), lithium chloride (LiCl), cadmium sulfide (CdS), nickel hydroxide (Ni(OH)_2_), calcium hydroxide (Ca(OH)_2_), calcium silicate (Ca₂O₄Si), vanadium phosphate, triiron tetraoxide (Fe_3_O_4_)
Material class “metal”: copper, silver, nickel, gold, aluminum, zirconium, platinum, palladium, chromium, aluminum
Material class “organic”: polyethylene, epoxy, polystyrene, polyaniline (PANi), cellulose, polyester, polyvinyl alcohol (PVA), polyurethane, polypropylene, polypyrrole, chitosan, polymethylmethacrylate (PMMA), nylon, polycarbonate, polyamide, polyimide, hydrogel, collagen, polyelectrolyte, pyridine, glucose oxidase, polyacrylonitrile, polyvinylidene, polyvinyl chloride (PVC), polyvinylidene fluoride (PVDF), polysulfone, polyvinylpyrrolidone, polycaprolactone, polybutadiene, polylactic acid, polysaccharide, polydimethylsiloxane, chitin, polythiophene, cyclodextrin, polyacrylamide, polyolefin, vinylpyridine, carboxymethyl cellulose, polylactide, polyetherimide, polydopamine

The number of scientific papers with the top 10 and top 100 most frequently occurring material words are shown in Table 3 and S1 Table, respectively. This number differed widely among the various material words; graphene showed the maximum occurrence number at 8736, and calcium hydroxide showed the minimum occurrence number at 36. The top 10 most frequently occurring material words comprised 3 carbon, 2 ceramic, 2 metal, and 3 organic materials. Although carbon materials accounted for a small proportion of the 100 material words, most of them appeared in many scientific papers.

**Table 3:** Number of scientific papers in which each of the top 10 most commonly occurring material words appeared.

Material word	Number of papers in which the word occurred	Material word	Number of papers in which the word occurred
graphene	8736	aluminum	3500
epoxy	7980	cellulose	3359
CNT	6386	graphite	3269
SiO_2_	5741	PANi	2827
TiO_2_	4382	copper	2475

### Counting the co-occurrence between two material words

We counted the number of material words that co-occurred with the 100 material words in 2012 or 2015, and we named these material words “co-occurring material words”. The numbers of co-occurring material words of the top 10 and top 100 most commonly occurring material words are shown in Table 4 and S2 Table, respectively. This number varied greatly depending on the material words, as the maximum and minimum number of co-occurring words in 2015 were 85 for silica and 2 for vanadium phosphate.

**Table 4:** Number of co-occurring material words and the percentage of co-occurrence of the top 10 most commonly occurring material words.

Material word	Number of co-occurring material words		Percentage of co-occurrence	Material word	Number of co-occurring material words		Percentage of co-occurrence
	2012	2015			2012	2015	
graphene	43	80	66.1%	aluminum	57	62	11.9%
epoxy	62	72	27.0%	cellulose	50	60	20.4%
CNT	76	82	26.1%	graphite	64	73	25.7%
SiO_2_	78	85	33.3%	PANi	54	65	24.4%
TiO_2_	63	70	19.4%	copper	52	67	31.9%

We calculated the average percentages of co-occurrence in the material word pairs for each material class and compared these values (Table 5). The percentages of co-occurrence with the 100 material words in each material class for carbon, ceramic, metal, and organic materials were 56.3%, 34.5%, 56.8%, and 39.0%, respectively. The percentage of co-occurrence of the carbon and metal material classes was high (85.7%), whereas that of the ceramic and organic material classes was low (29.6%).

**Table 5:** Percentage of co-occurrence in material word pairs with each material class.

Material class	Carbon	Ceramic	Metal	Organic	100 material words
**Carbon**	83.7%	50.2%	85.7%	50.7%	56.3%
**Ceramic**	50.2%	33.6%	47.8%	29.6%	34.5%
**Metal**	85.7%	47.8%	86.0%	53.8%	56.8%
**Organic**	50.7%	29.6%	53.8%	42.9%	39.0%

### Link prediction of a co-occurrence network

To identify the conditions with high link prediction accuracies, we conducted link prediction for 24 patterns (the patterns were combinations of eight network indexes and three training periods) and compared the results. The AUCs and average accuracies of link prediction in the patterns are shown in S3 Table, and the ROCs are shown in S1–S8 Figs.

By comparing the results for each network index, it was seen that the network indexes showed higher values with CN or CNSH. The average AUCs using CN, JC, RA, AA, PA, CNSH, RASH and WIC were 77.0%, 70.7%, 74.8%, 73.9%, 71.8%, 77.3%, 57.2% and 55.0%, respectively. The average accuracies using CN, JC, RA, AA, PA, CNSH, RASH and WIC were 67.6%, 65.2%, 67.8%, 67.8%, 66.4%, 68.8%, 57.4% and 55.8%, respectively. Since the values with CN and CNSH were high, CN and CNSH were regarded as the appropriate network indexes for this link prediction technique.

By comparing the results for each training period, it was determined that the training period with the highest values depended on the network index. The average AUC values using TRP1, TRP2, and TRP3 were 69.6%, 69.9%, and 69.6%, respectively; these values were nearly the same. However, the training period with the highest AUC differed according to the network index; for example, link prediction using CN and CNSH showed the highest values with TRP2 and TRP1, respectively. The average accuracies using TRP1, TRP2 and TRP3 were 63.9%, 65.1% and 64.8%, respectively; these values were nearly the same. Nevertheless, the training period with the highest average accuracy differed according to the network index; for example, link prediction using CN and CNSH showed the highest values with TRP3 and TRP1, respectively. Since the training period with the highest values differed according to the network index as above, the best training period for the link prediction depended on the network index.

From the above results, the following pairs of network indexes and training periods with higher AUCs and average accuracies were determined to be appropriate for link prediction in this study: {CN, TRP2}, {CN, TRP3}, and {CNSH, TRP1}. To evaluate the usefulness of these pairs, we conducted link prediction during the testing period and calculated the accuracies with these pairs. First, we counted the TPs, FPs, FNs, and TNs of each cutoff with CN and CNSH and calculated the sensitivities, specificities and average accuracies (Tables 6 and 7). Next, we conducted link prediction with the following three cutoffs (defined as positive): CN ≥ 9, CN ≥ 5, and CNSH ≥ 8, which were calculated using {CN, TRP2}, {CN, TRP3}, and {CNSH, TRP1}, respectively; since the average accuracies of link prediction using each pair were 64.7%, 64.1% and 56.9%, respectively, link prediction using {CN, TRP2} showed the highest accuracy.

**Table 6:** Accuracies of link prediction in the testing periods for each cutoff using CN.

Cutoff	Sensitivity	Specificity	Average accuracy
≥1	658/666 (98.8%)	508/6736 (7.5%)	53.2%
≥2	648/666 (97.3%)	1274/6736 (18.9%)	58.1%
≥3	608/666 (91.3%)	1938/6736 (28.8%)	60.0%
≥4	568/666 (85.3%)	2632/6736 (39.1%)	62.2%
≥5	536/666 (80.5%)	3214/6736 (47.7%)	64.1%
≥6	480/666 (72.1%)	3780/6736 (56.1%)	64.1%
≥7	444/666 (66.7%)	4240/6736 (62.9%)	64.8%
≥8	402/666 (60.4%)	4640/6736 (68.9%)	64.6%
≥9	364/666 (54.7%)	5036/6736 (74.8%)	64.7%
≥10	316/666 (47.4%)	5380/6736 (79.9%)	63.7%

**Table 7:** Accuracies of link prediction in the testing periods for each cutoff using CNSH.

Cutoff	Sensitivity	Specificity	Average accuracy
≥1	649/666 (0.974%)	469/6736 (0.93)	52.2%
≥2	624/666 (0.937%)	1065/6736 (0.842)	54.8%
≥3	575/666 (0.863%)	1678/6736 (0.751)	55.6%
≥4	536/666 (0.805%)	2231/6736 (0.669)	56.8%
≥5	496/666 (0.745%)	2675/6736 (0.603)	57.1%
≥6	454/666 (0.682)	3111/6736 (0.538)	57.2%
≥7	410/666 (0.616)	3542/6736 (0.474)	57.1%
≥8	369/666 (0.554)	3936/6736 (0.416)	56.9%
≥9	333/666 (0.5)	4261/6736 (0.367)	56.6%
≥10	305/666 (0.458)	4555/6736 (0.324)	56.7%

CN ≥ 7 showed the highest average accuracy (64.8%) of cutoffs using CN; this value was close to the average accuracy using CN ≥ 9 (64.7%). Therefore, {CN, TRP2} showed a high link prediction accuracy during the testing period. In the testing period, link prediction using CN≥9 successfully predicted the co-occurrence of 364 of 666 co-occurrence material word pairs, and it successfully predicted the non-co-occurrence of 5036 of 6736 non-co-occurrence material word pairs. Link prediction with CN≥9 showed lower sensitivity (54.7%) and higher specificity (74.8%) than that using CN≥7, of which the sensitivity and specificity were 66.7% and 62.9%, respectively. In agreement with the previous results, {CN, TRP2} was regarded as an appropriate pair for link prediction during the testing period.

## Discussion

### Listing material words from collected papers

Even though carbon accounts for a small proportion of the 100 material words, most of them appear in many scientific papers. This observation implies that carbon materials exhibit high levels of usefulness as components of composites. For example, graphene, which appears in most papers mentioning some of the 100 material words, is composed of many kinds of materials, including organic, ceramic and metal materials, and its wide-ranging applications include battery electrode materials, reinforced plastics, photocatalytic materials, and cell culture basics [39–42]. CNTs, which appear in the third most papers out of the 100 material words, are compounded with many kinds of materials, including ceramic, metal and organic materials [43, 44]. The applications of CNT composites vary; for example, CNT composites have been applied in reinforced plastics, electrode materials and wearable devices [45]. In addition, CNT composite development is expected to accelerate in the future because of the establishment of mass production methods for CNTs [46]. In contrast, calcium hydroxide, which occurs in the fewest scientific papers of the 100 material words, is compounded mainly with resin. The application range of calcium hydroxide is narrower than those of graphene and CNTs, as calcium hydroxide-based compounds are primarily used for crown restoration [47].

Prof. Bunshi Fugetsu of the University of Tokyo, who is an expert in composite materials, claimed that this result represents the attractiveness of carbon materials. Graphene and CNTs have high reactivity and strength as components of composites. Plus, many composites can only be realized with them because of their unique shape.

### Counting co-occurrence instances between two material words

We test our research hypothesis that material word pairs co-occurring for the first time were reported as composite materials. We investigate whether material word pairs that co-occurred with silica or CNTs for the first time from 2012 to 2015 are described as composite materials. As a result, 11 out of 13 material word pairs are actually reported as composites: {silica, carboxymethyl cellulose}, {silica, polyetherimide}, {silica, polyolefin}, {silica, PVDF}, {silica, LiFePO_4_}, {CNT, zirconium}, {CNT, polydopamine}, {CNT, C_3_N_4_}, {CNT, CoFe_2_O4}, {CNT, CuO} and {CNT, Li_3_V_2_} [48–58]. The others are not reported as composites: {silica, kaolinite} and {silica, MoS_2_} [59, 60]. Since most pairs of materials are composited, our hypothesis is reasonable.

As a result of our method, the number of co-occurring material words varies widely. This phenomenon occurs partly because the ease of handling is different in each material. For example, graphene and CNTs are manageable due to their high reactivity and strength, but fullerene is not manageable because of its poor solubility [61]. Fullerene is relatively uncommon as a research theme because research on fullerene is likely to take more time to make discoveries. However, Prof. Fugetsu states that fullerene is a very interesting material and is worth to be researching.

### Link prediction of a co-occurrence network

The cutoff calculated from {CN, TRP2} shows high accuracy for link prediction in both the training period and the testing period. We discuss the cause of this result below.

First, we examined why CN and CNSH showed high link prediction accuracy in the training periods. We concluded that this phenomenon occurs because CN and CNSH have similar properties to the created co-occurrence networks of material words. CN and CNSH show that two nodes having a link to an in-common node are more likely to have a link than those without links to in-common nodes. Nodes in the created co-occurrence networks of material words that are linked to many in-common nodes tend to have new links (details are described in the next subsection). Nodes with many neighbors tend to have many common neighbors with another node. In other words, link prediction using CN or CNSH is more likely to correctly predict new links, and it shows high accuracies and AUCs. However, RA, AA, and RASH are based on the theory that two nodes linked to a node with few in-common neighbors are likely to have a link. Thus, link prediction using these indexes tends to show that nodes linked to few in-common neighbors have new links in the material word co-occurrence network, even though nodes with many neighbors are more likely to have new links. RA, AA, and RASH show lower accuracies and AUCs than CN and CNSH because their nodes linked to few in-common nodes do not have new links.

Next, we discuss why link prediction using CNSH shows low average accuracies and AUCs in the testing period. This result indicates that adding community information (in this case, material class) reduces the link prediction accuracy; in other words, the instances of co-occurrence between two material words in different material classes increase. The number of material words in which graphene and CNTs co-occurred in the testing period increases rapidly from 43 to 93 and from 76 to 91, respectively. Since only five of the 100 material words are carbon materials, 95 of the 100 material words are noncarbon materials; in other words, graphene and CNTs co-occur with many material words in other material classes.

Finally, we infer why the optimal training period differs by network index. We assume that this is in part because the instances of co-occurrence between material words in different classes increase rapidly. Link prediction shows high accuracy in TRP1 in the case of using network indexes that add community information (CNSH, RASH, and WIC); however, it tends to exhibit low accuracy in TRP1 when using network indexes that do not add community information (CN, JC, RA, AA, and PA). This result indicates that linking old data (in this case, TRP1) is optimal when using network indexes that add community information, such as CNSH, because there are few instances of co-occurrence between two material words in different material classes in old data. As above, we conclude that network indexes that do not add community information are appropriate for new data, and other indexes are appropriate for old data.

### Evaluation of the link prediction accuracy

The cutoff calculated from {CN, TRP2} is defined as the best in our method because it shows high accuracy in both the training and testing periods. This cutoff shows high specificity (74.8%) during the training period and is regarded as a useful cutoff for the following reasons. Values evaluating the accuracy of true/false predictions depend on the case. In the case of cancer screening, sensitivity is more important than specificity because positive cases (i.e., patients who have cancer) must not be overlooked. In contrast, cold assessment emphasizes specificity because it is important to reduce false positives (i.e., patients who do not have a cold but are diagnosed with it) to accelerate examination. We consider specificity to be more important than sensitivity in our method because researchers need to avoid research themes that are unlikely to produce results due to time and budget limitations. This finding is also because an emphasis on sensitivity may lead to a focus on research themes that are only likely to produce results, decelerating innovation. Since innovation is based on diverse knowledge, researchers must broaden their research scope to avoid disregarding innovative discoveries [62]. From the aforementioned theories, we consider the cutoff calculated from {CN, TRP2} to be useful, indicating that we have succeeded in calculating a useful cutoff with the testing data.

Next, we analyze the common characteristics of material word pairs for which link prediction with the best cutoff cannot predict co-occurrence. The ratio of FN material pairs for each material word is calculated from the following formula, and we determine the material words of the co-occurrence that were overlooked based on the ratio.


$$A=\frac{B⋂C}{B}$$ (5)

*A*: FN rate of material word pairs for the material word *x*, *B*: Material word pairs containing x that co-occur during TRP2, *C*: Material word pairs that are not predicted to co-occur.

As a result, the FN of the following 12 material words is 100%: carboxymethyl cellulose, polydopamine, V_2_O_5_, polylactide, C_3_N_4_, Li_3_V_2_, MoO_3_, cyclodextrin, melamine, CaCl_2_, polyetherimide and LiCl. These material words tended to co-occur with a few material words in 2012, as cyclodextrin co-occurred with fewer than 10 material words. On the other hand, the FNs of the following material words were 0%: gold, PVA, polyurethane, aluminum, PMMA, BaTiO_3_, nylon, polyimide, B_4_C and titanium diboride (TiB_2_). As they co-occur with more than 10 material words, they tend to have co-occurrence with more words than those for which FN is 100%. Therefore, our method is likely to show higher accuracy for predicting co-occurrence with material words that already co-occur with many words.

Because material words that co-occur with many words are more likely to have new instances of co-occurrence, we assume that material words that co-occur with those already having many co-occurring words can be predicted by focusing on non-co-occurring words. To verify this, we calculate the percentage of co-occurrence from 1/1/2016 to 8/15/2020 of the 100 material words, and the values of the top 10 most frequently occurring material words. The 100 material words are shown in Table 8 and S4 Table, respectively. As the number of co-occurring material words in 2015 and the co-occurrence rate between 2016 and 2020 show a high correlation coefficient of 0.732, we find that in general, the greater the number of co-occurring material words is, the higher the percentage of co-occurrence. While the average of 100 material words is 14.1%, only graphene and CNTs, which co-occur with more than 80 material words in 2015, show a high co-occurrence rate exceeding 50%. From the above results, the prediction of the co-occurrence of material words that already have many instances of co-occurrence is highly possible by focusing on the material words that are not unreasonable.

**Table 8:** Percentage of the co-occurrence of the top 10 most frequently occurring material words.

Material word	Percentage of co-occurrence from 1/1/2016 to 8/15/2020	Material word	Percentage of co-occurrence from 1/1/2016 to 8/15/2020
graphene	68.4%	aluminum	32.4%
epoxy	18.5%	cellulose	41.0%
CNT	52.9%	graphite	30.8%
SiO_2_	21.4%	PANi	32.4%
TiO_2_	31.0%	copper	37.5%

## Conclusions

Innovative material combinations were emphasized. However, it was difficult for previous methods to detect possible pairs of materials from a wide range of materials because the physical properties of some materials such as polymers were hard to quantify. To solve this problem, we predicted possible pairs of materials by conducting link prediction on the co-occurrence network under the assumption that pairs of material words co-occurring in scientific papers on composites are reported as composite materials. Our MI method analyzed various kinds of materials including polymer materials such as cellulose and succeeded in searching compoundable material combinations from thousands of pairs, which was far more than those in previous studies [10–12]. Our method exhibited the potential to promote compoundable and innovative pairs of materials by the cross-sectional exploration of materials.

The limitation of our method was that its specificity and prediction accuracy of material words that co-occurred with fewer others were low. Thus, our future work is to conduct link prediction on only material words co-occurring with fewer others to find better conditions. In addition, we plan to try other network indexes and/or implement multiple indexes to improve the sensitivity and specificity.

## Supporting information

S1 Fig ROC curves for CN with each training period. (TIFF)

S2 Fig ROC curves for JC with each training period. (TIFF)

S3 Fig ROC curves for RA with each training period. (TIFF)

S4 Fig ROC curves for AA with each training period. (TIFF)

S5 Fig ROC curves for PA with each training period. (TIFF)

S6 Fig ROC curves for CNSH with each training period. (TIFF)

S7 Fig ROC curves for RASH with each training period. (TIFF)

S8 Fig ROC curves for WIC with each training period. (TIFF)

S1 Table The number of scientific papers in which each of the 100 material words occurred. (DOCX)

S2 Table 100 material words and the number of papers in which they were found. (DOCX)

S3 Table The number of co-occurring material word(s) and the percentage of co-occurrence of 100 material word(s). (DOCX)

S4 Table The number of scientific papers in which each of the 100 material words occurred. (DOCX)

S1 Data (ZIP)

## References

[1] Mehra A. Composites Market Worth $126.3 Billion by 2026. 2021 [cited 3 February 2023]. Available from: https://www.marketsandmarkets.com/PressReleases/composite.asp.

[2] Meguid SA, Sun Y. On the tensile and shear strength of nano-reinforced composite interfaces. Mater Design. 2004;25: 289–296.

[3] Vipin AK, Fugetsu B, Sakata I, Isogai A, Endo M, Li M, et al. Cellulose nanofiber backboned Prussian blue nanoparticles as powerful adsorbents for the selective elimination of radioactive cesium. Sci Rep. 2016;6: 37009. doi: 10.1038/srep37009 27845441 PMC5109467

[4] Wang Y, Fugetsu B, Wang Z, Gong W, Sakata I, Morimoto S, et al. Nitrogen-doped porous carbon monoliths from polyacrylonitrile (PAN) and carbon nanotubes as electrodes for supercapacitors. Sci Rep. 2017;7: 40259. doi: 10.1038/srep40259 28074847 PMC5225489

[5] Takeuchi K, Takizawa Y, Kitazawa H, Fujii M, Hosaka K, Ortiz-Medina J, et al. Salt rejection behavior of carbon nanotube-polyamide nanocomposite reverse osmosis membranes in several salt solutions. Desalination. 2018;443: 165–171.

[6] Kitano H, Takeuchi K, Ortiz-Medina J, Cruz-Silva R, Morelos-Gomez A, Fujii M, et al. Enhanced antifouling feed spacer made from a carbon nanotube-polypropylene nanocomposite. ACS Omega. 2019;4: 15496–15503. doi: 10.1021/acsomega.9b01757 31572850 PMC6761618

[7] Rajan K. Materials informatics. Mater Today. 2012;15: 470.

[8] MatWeb. Online Materials Information Resource—MatWeb. 1996 [cited 3 February 2023]. Available from: https://www.matweb.com/.

[9] Rodgers JR, Cebon D. Materials informatics. MRS Bull. 2006;31: 975–980.

[10] Yu G, Chen J, Zhu L. Data mining techniques for materials informatics: datasets preparing and applications. In: 2009 second international symposium on knowledge acquisition and modeling. Wuhan, China: IEEE; 2009. pp. 189–192.

[11] Hassan AM, Alrashdan A, Hayajneh MT, Mayyas AT. Prediction of density, porosity and hardness in aluminum–copper-based composite materials using artificial neural network. J Mater Process Technol. 2009;209: 894–899.

[12] Doreswamy H, Vanajaskhi MN. Similarity measuring approach for engineering materials selection. Int J Comput Intell Syst. 2010;3: 115–122.

[13] Mo Y, Ong SP, Ceder G. First principles study of the Li_10_GeP_2_S_12_ lithium super Ionic conductor material. Chem Mater. 2011;24: 15–17.

[14] N Adams. Polymer Informatics. Adv Polym Sci. 2010;225: 107–149.

[15] Tshitoyan V, Dagdelen J, Weston L, Dunn A, Rong Z, Kononova O, et al. Unsupervised word embeddings capture latent knowledge from materials science literature. Nature. 2019;571: 95–98. doi: 10.1038/s41586-019-1335-8 31270483

[16] Rzhetsky A, Foster JG, Foster IT, Evans J. Choosing experiments to accelerate collective discovery. Proc Natl Acad Sci U S A. 1/1/20152: 14569–14574. 10.1073/pnas.1509757112 PMC4664375 26554009

[17] Sha R, Komori K, Badhulika S. Graphene–polyaniline composite based ultra-sensitive electrochemical sensor for non-enzymatic detection of urea. Electrochim Acta. 2017;233: 44–51.

[18] Trapalis A, Todorova N, Giannakopoulou T, Boukos N, Speliotis T, Dimotikali D, et al. TiO2/graphene composite photocatalysts for NOx removal: a comparison of surfactant-stabilized graphene and reduced graphene oxide. Appl Catal B Environ. 2016;180: 637–647.

[19] Sasaki H, Sakata I. Identifying potential technological spin-offs using hierarchical information in international patent classification. Technovation. 2021;100: 102192.

[20] Lande D, Fu M, Guo W, Balagura I, Gorbov I, Yang H. Link prediction of scientific collaboration networks based on information retrieval. World Wide Web. 2020;23: 2239–2257.

[21] Marcotte EM, Pellegrini M, Ng HL, Rice DW, Yeates TO, Eisenberg D. Detecting protein function and protein-protein interactions from genome sequences. Science. 1999;285: 751–753. doi: 10.1126/science.285.5428.751 10427000

[22] Kashima H, Abe N. A parameterized probabilistic model of network evolution for supervised link prediction. In: Sixth international conference on data mining (ICDM’06). Hong Kong, China: IEEE; 2006. pp. 340–349.

[23] Newman MEJ. Clustering and preferential attachment in growing networks. Phys Rev E. 2001;64: 025102. doi: 10.1103/PhysRevE.64.025102 11497639

[24] Jaccard P. Etude de la distribution florale dans une portion des Alpes et du Jura. Bull Soc Vaud Sci Nat. 1901;37: 547–579.

[25] Zhou T, Lü L, Zhang YC. Predicting missing links via local information. Eur Phys J B. 2009;71: 623–630.

[26] Adamic LA, Adar E. Friends and neighbors on the web. Soc Netw. 2003;25: 211–230.

[27] Liben-Nowell D, Kleinberg J. The link prediction problem for social networks. In: Proceedings of the twelfth international conference on information and knowledge management. New York, NY, USA: ACM; 2003. pp. 556–559.

[28] Innovation Policy Research Center, The University of Tokyo, and Kajikawa Laboratory, Graduate School of Innovation Management, Tokyo Institute of Technology. Academic Landscape System. 2010 [cited 3 February 2023]. Available from: https://academic-landscape.com/.

[29] Dunlavy DM, Kolda TG, Acar E. Temporal link prediction using matrix and tensor factorizations. ACM Trans Knowl Discov Data. 2011;5: 1–27.

[30] Acar E, Dunlavy DM, Kolda TG. Link prediction on evolving data using matrix and tensor factorizations. In: 2009 IEEE international conference on data mining workshops. Miami, FL, USA: IEEE; 2009. pp. 262–269.

[31] Bornmann L, Mutz R. Growth rates of modern science: a bibliometric analysis based on the number of publications and cited references. J Assoc Inf Sci Technol. 2015;66: 2215–2222.

[32] Cao RM, Liu SY, Xu XK. Network embedding for link prediction: the pitfall and improvement. Chaos Interdiscip J Nonlinear Sci. 2019;29: 103102. doi: 10.1063/1.5120724 31675842

[33] Bai M, Hu K, Tang Y. Link prediction based on a semi-local similarity index. Chin Phys B. 2011;20: 128902.

[34] Soundarajan S, Hopcroft J. Using community information to improve the precision of link prediction methods. In: Proceedings of the 21st international conference on world wide web. New York, NY, USA: ACM; 2012. pp. 607–608.

[35] Valverde-Rebaza JC, Lopes ADA. Link prediction in complex networks based on cluster information. In: Barros LN, Finger M, Pozo AT, Gimenénez-Lugo GA, Castilho M, editors. Advances in artificial intelligence—SBIA 2012. Berlin, Heidelberg: Springer; 2012. pp. 92–101.

[36] Yerushalmy J. Statistical problems in assessing methods of medical diagnosis, with special reference to X-Ray techniques. Public Health Rep (1896–1970). 1947;62: 1432–1449. 20340527

[37] Metz CE. Basic principles of ROC analysis. Semin Nucl Med. 1978;8: 283–298. doi: 10.1016/s0001-2998(78)80014-2 112681

[38] Bradley AP. The use of the area under the ROC curve in the evaluation of machine learning algorithms. Pattern Recognit. 1997;30: 1145–1159.

[39] Wu ZS, Zhou G, Yin LC, Ren W, Li F, Cheng HM. Graphene/metal oxide composite electrode materials for energy storage. Nano Energy. 1/1/201207–131.

[40] Liu S, Yan H, Fang Z, Wang H. Effect of graphene nanosheets on morphology, thermal stability and flame retardancy of epoxy resin. Compos Sci Technol. 2014;90: 40–47.

[41] Zhang H, Lv X, Li Y, Wang Y, Li J. P25-graphene composite as a high performance photocatalyst. ACS Nano. 2009;4: 380–386. 10.1021/nn901221k 20041631

[42] Ding X, Liu H, Fan Y. Graphene-based materials in regenerative medicine. Adv Healthc Mater. 2015;4: 1451–1468. doi: 10.1002/adhm.201500203 26037920

[43] Coleman JN, Khan U, Blau WJ, Gun’ko YK. Small but strong: a review of the mechanical properties of carbon nanotube–polymer composites. Carbon. 2006;44: 1624–1652.

[44] Bakshi SR, Lahiri D, Agarwal A. Carbon nanotube reinforced metal matrix composites—a review. Int Mater Rev. 2010;55: 41–64.

[45] Coleman JN, Khan U, Gun’ko YK. Mechanical reinforcement of polymers using carbon nanotubes. Adv Mater. 2006;18: 689–706.

[46] National Institute of Advanced Industrial Science and Technology (AIST). World’s First Super-Growth Carbon Nanotube Mass Production Plant Opens. 2016 [cited 3 February 2023]. Available from: https://www.aist.go.jp/aist_e/list/latest_research/2016/20160524/en20160524.html.

[47] Schuurs AHB, Gruythuysen RJM, Wesselink PR. Pulp capping with adhesive resin-based compositevs.calcium hydroxide: a review. Dent Traumatol. 2000;16: 240–250. doi: 10.1034/j.1600-9657.2000.016006240.x 11202889

[48] Somodi F, Kong CS, Santos JC, Morse DE. Vesicular hydrogen silsesquioxane-mediated synthesis of nanocrystalline silicon dispersed in a mesoporous silica/suboxide matrix, with potential for electrochemical applications. New J Chem. 2015;39: 621–630.

[49] Romero AI, Parentis ML, Habert AC, Gonzo EE. Synthesis of polyetherimide/silica hybrid membranes by the sol–gel process: influence of the reaction conditions on the membrane properties. J Mater Sci. 2011;46: 4701–4709.

[50] Gharehbas N, Shakeri A. Preparation and thermal and physical properties of nano-silica modified and unmodified. Orient J Chem. 2015;31: 207–212.

[51] Amanieu HY, Rosato D, Sebastiani M, Massimi F, Lupascu DC. Mechanical property measurements of heterogeneous materials by selective nanoindentation: application to LiMn2O4 cathode. Mater Sci Eng A. 2014;593: 92–102.

[52] Svitan’ko A, Scopets V, Novikova S, Yaroslavtsev A. The effect of composite formation with oxides on the ion conductivity of NASICON-Type LiTi2(PO4)3 and olivine-type LiFePO4. Solid State Ion. 2015;271: 42–47.

[53] Chu H, Zhang Z, Liu Y, Leng J. Self-response multi-functional composite material base on carbon nanotube paper using deicing, flame retardancy, thermal insulation, and lightning-strike protection. In: Behavior and mechanics of multifunctional materials and composites 2015. San Diego, California, United States: SPIE; 2015. pp. 181–188.

[54] Wang X, Lee PS. A polydopamine coated polyaniline single wall carbon nanotube composite material as a stable supercapacitor cathode in an organic electrolyte. J Mater Res. 2015;30: 3575–3583.

[55] Hu S, Wang H, Wang F, Bai J, Zhang L, Kang X, et al. Practical preparation of carbon black/carbon nitride compounds and their photocatalytic performance. Bull Korean Chem Soc. 2015;36: 2527–2533.

[56] Zhao J, Xie Y, Yu C, Le Z, Zhong R, Qin Y, et al. Preparation and characterization of the graphene–carbon nanotube/CoFe2O4/polyaniline composite with reticular branch structures. Mater Chem Phys. 2013;142: 395–402.

[57] Abbas SM, Hussain ST, Ali S, Abbas F, Ahmad N, Ali N, et al. One-pot synthesis of a composite of monodispersed CuO nanospheres on carbon nanotubes as anode material for lithium-ion batteries. J Alloys Compd. 2013;574: 221–226.

[58] Qiao YQ, Tu JP, Mai YJ, Cheng LJ, Wang XL, Gu CD. Enhanced electrochemical performances of multi-walled carbon nanotubes modified Li3V2(PO4)3/C cathode material for lithium-ion batteries. J Alloys Compd. 2011;509: 7181–7185.

[59] Seifollahzadeh P, Kalantar M, Ghasemi SS. Structure-property relationships of mullite-SiC-Al_2_O_3_–ZrO_2_ composites developed during carbothermal reduction of aluminosilicate minerals. J Alloys Compd. 2015;647: 973–980.

[60] Duan A, Li T, Zhao Z, Liu B, Zhou X, Jiang G, et al. Synthesis of hierarchically porous L-KIT-6 silica–alumina material and the super catalytic performances for hydrodesulfurization of benzothiophene. Appl Catal B Environ. 2015;165: 763–773.

[61] Jiang G, Yang Y. Preparation and tribology properties of water-soluble fullerene derivative nanoball. Arab J Chem. 2017;10: S870–S876.

[62] Tavassoli S, Carbonara N. The role of knowledge variety and intensity for regional innovation. Small Bus Econ. 2014;43: 493–509.

