# Supplementary figures and images for: Detecting possible pairs of materials for composites using a material word co-occurrence network
Source: PLoS One. 2024 Jan 26;19(1):e0297361. doi: 10.1371/journal.pone.0297361 (PMC10817182; doi:10.1371/journal.pone.0297361)

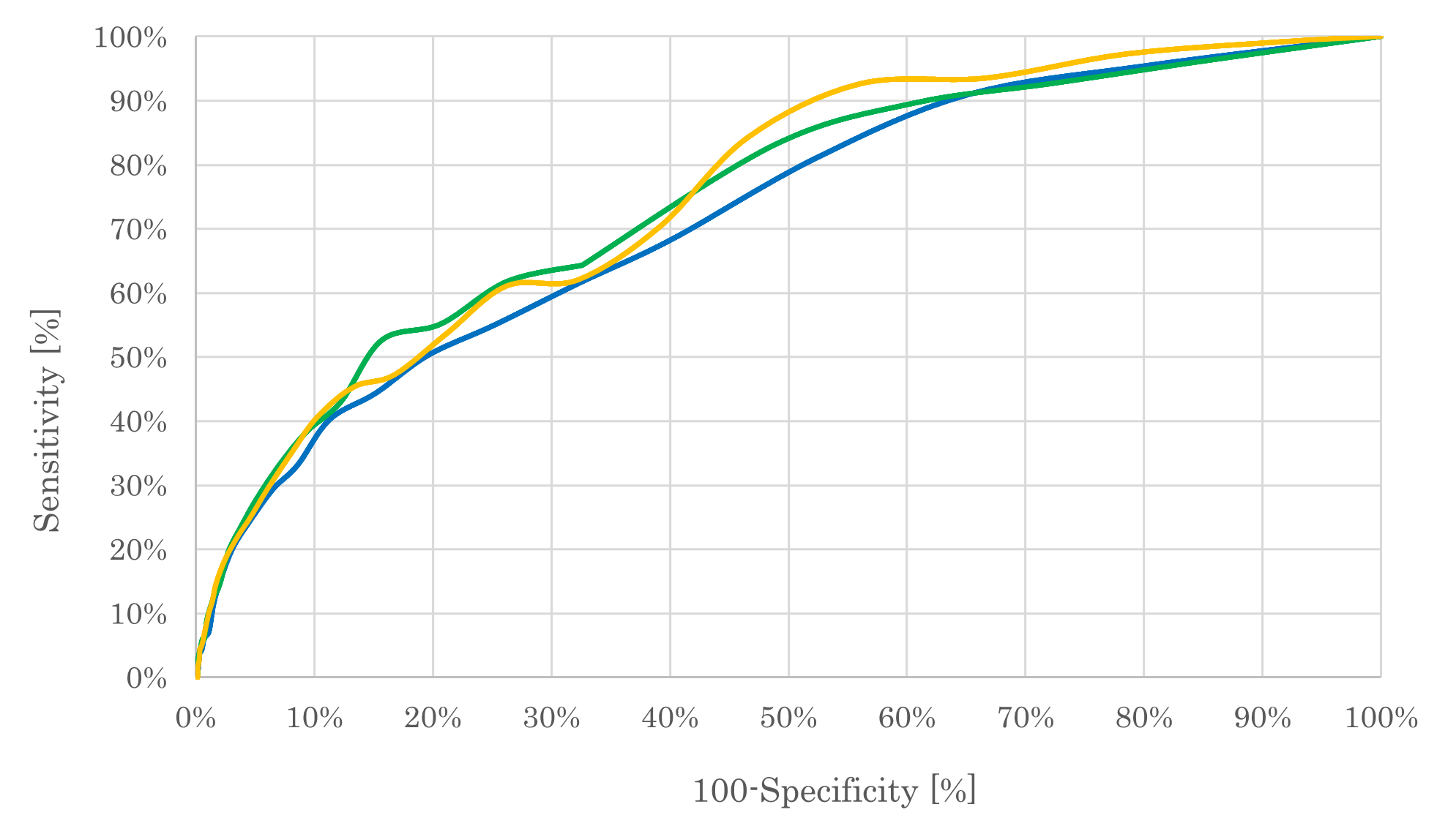

Supplement: S1 Fig — (TIFF) [file pone.0297361.s001.tiff]

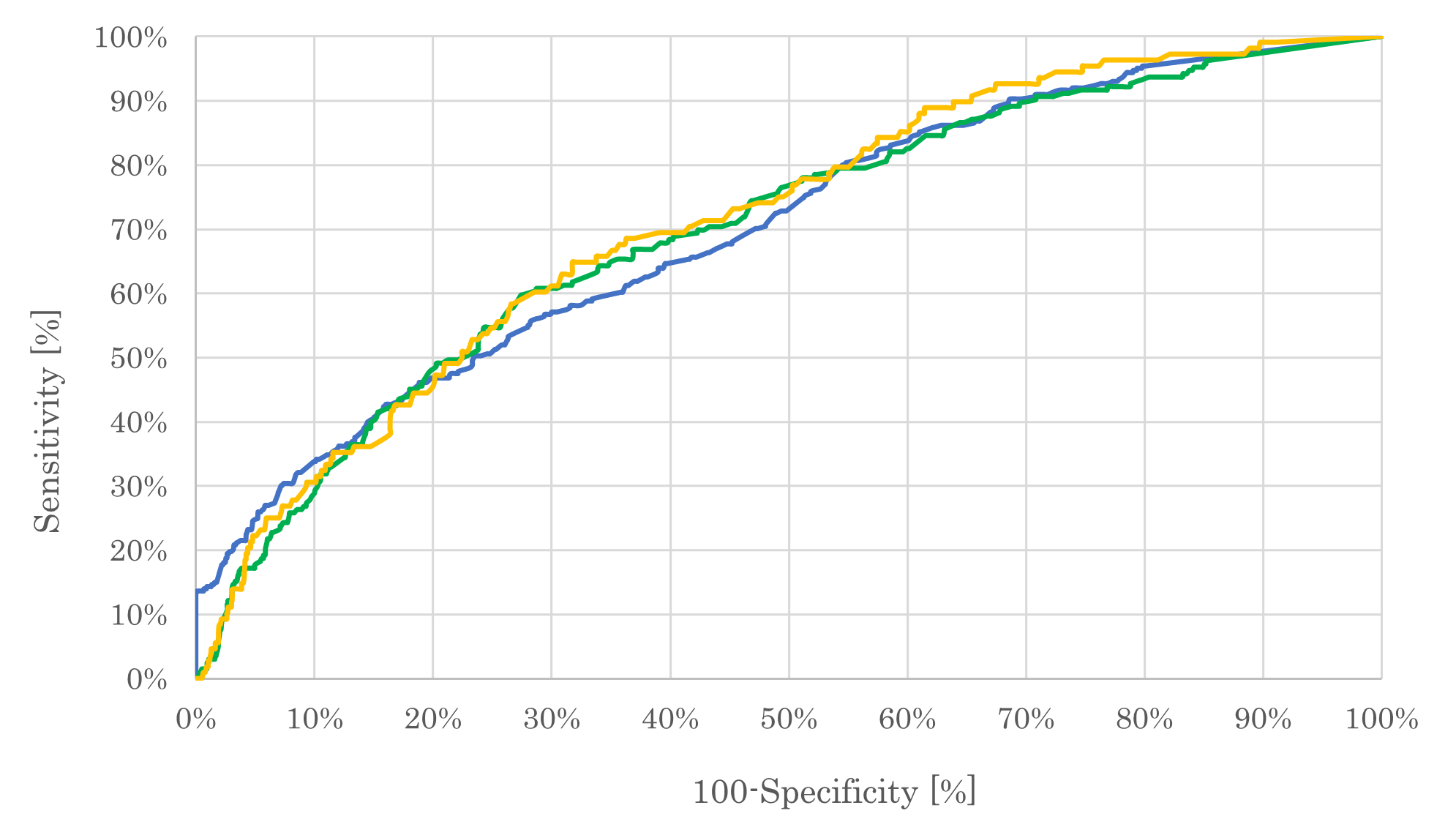

Supplement: S2 Fig — (TIFF) [file pone.0297361.s002.tiff]

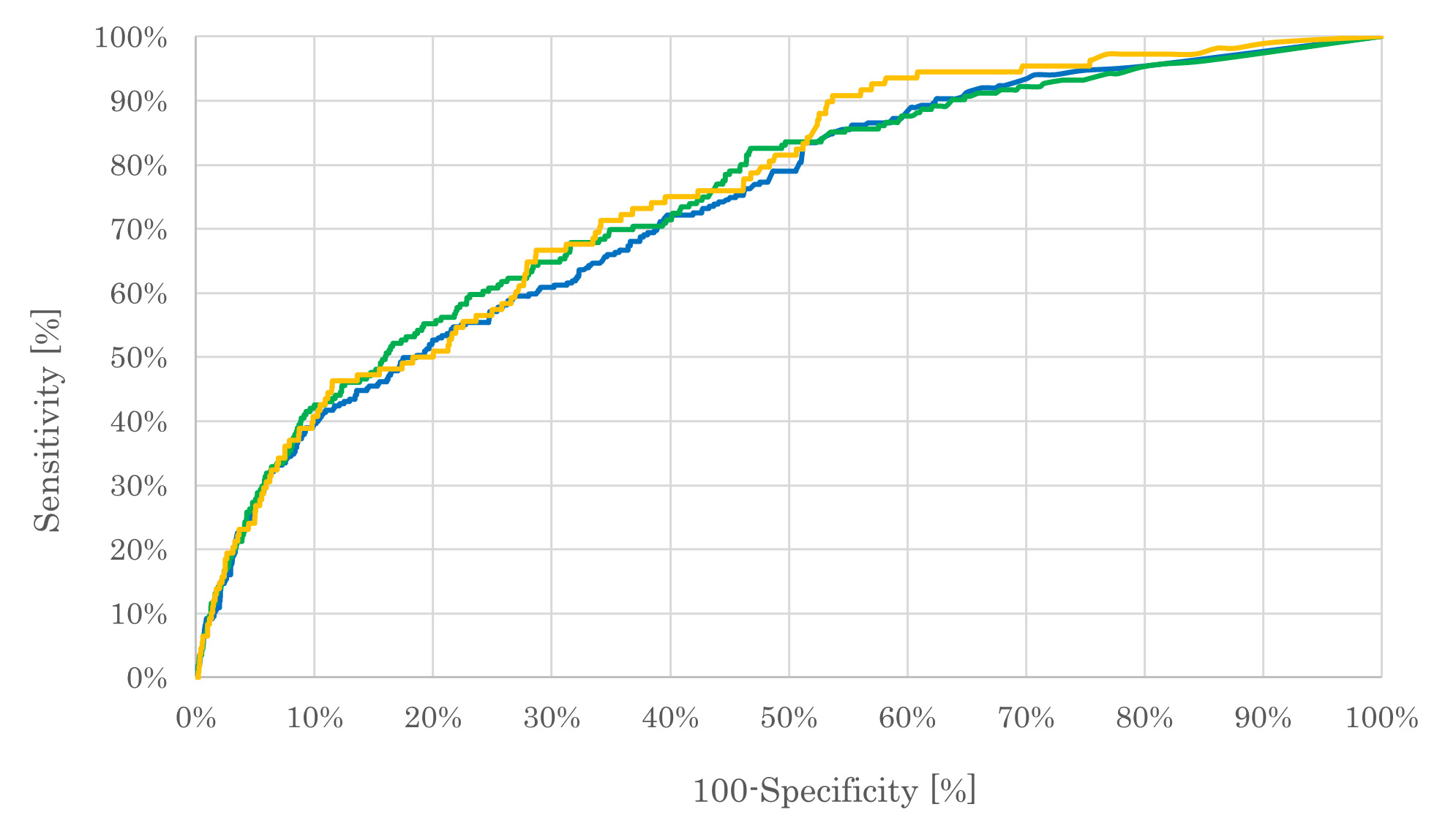

Supplement: S3 Fig — (TIFF) [file pone.0297361.s003.tiff]

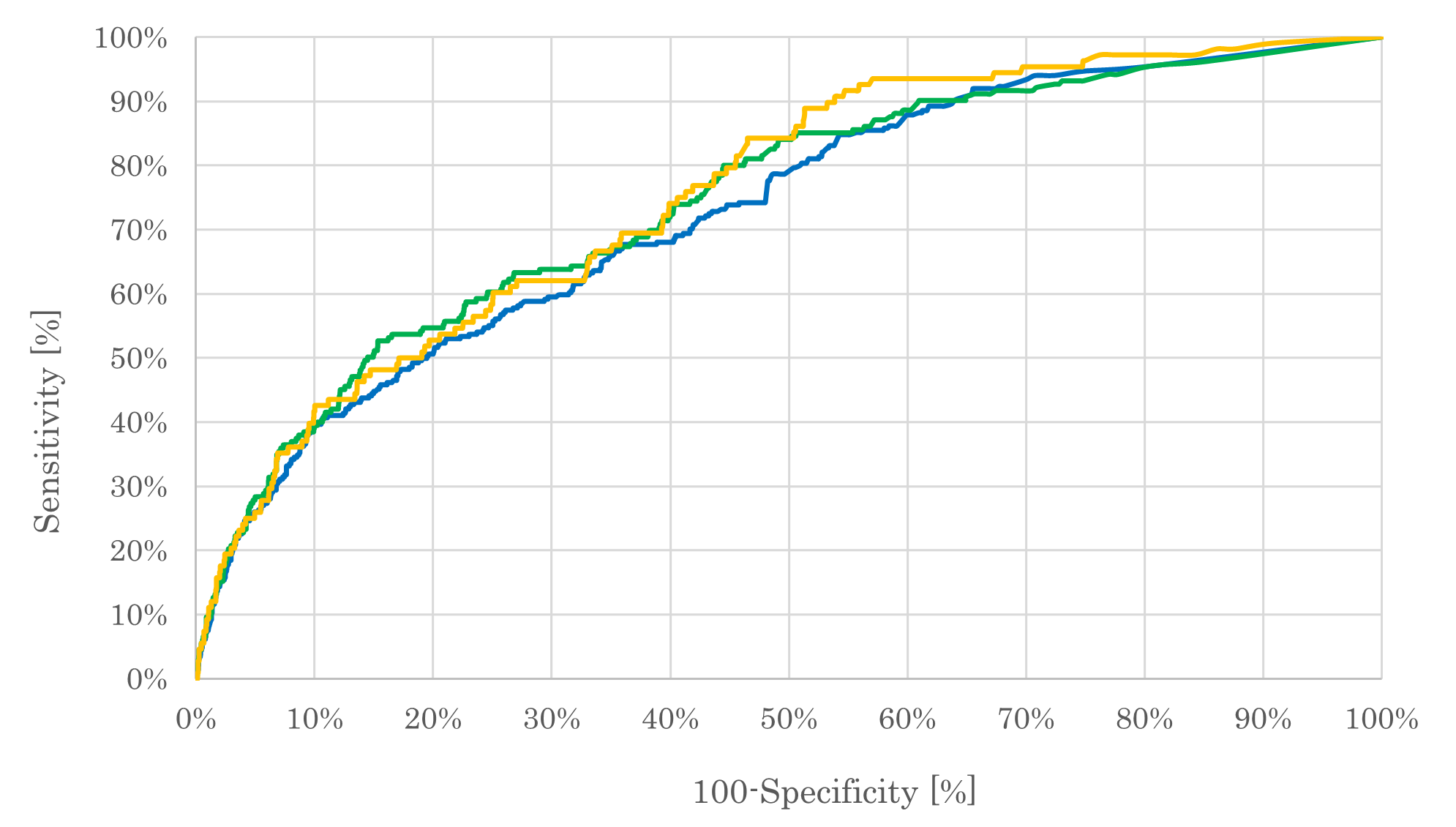

Supplement: S4 Fig — (TIFF) [file pone.0297361.s004.tiff]

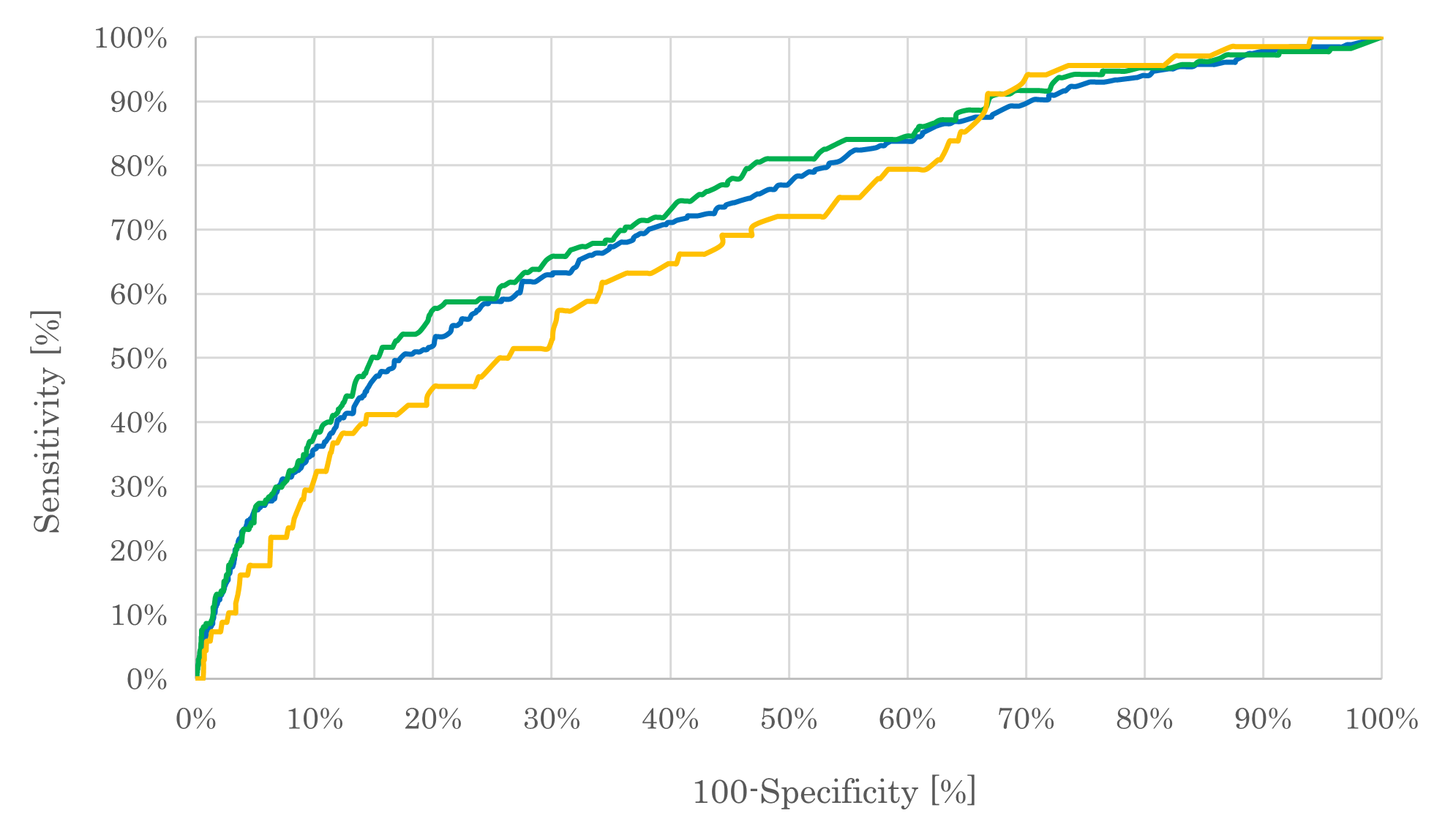

Supplement: S5 Fig — (TIFF) [file pone.0297361.s005.tiff]

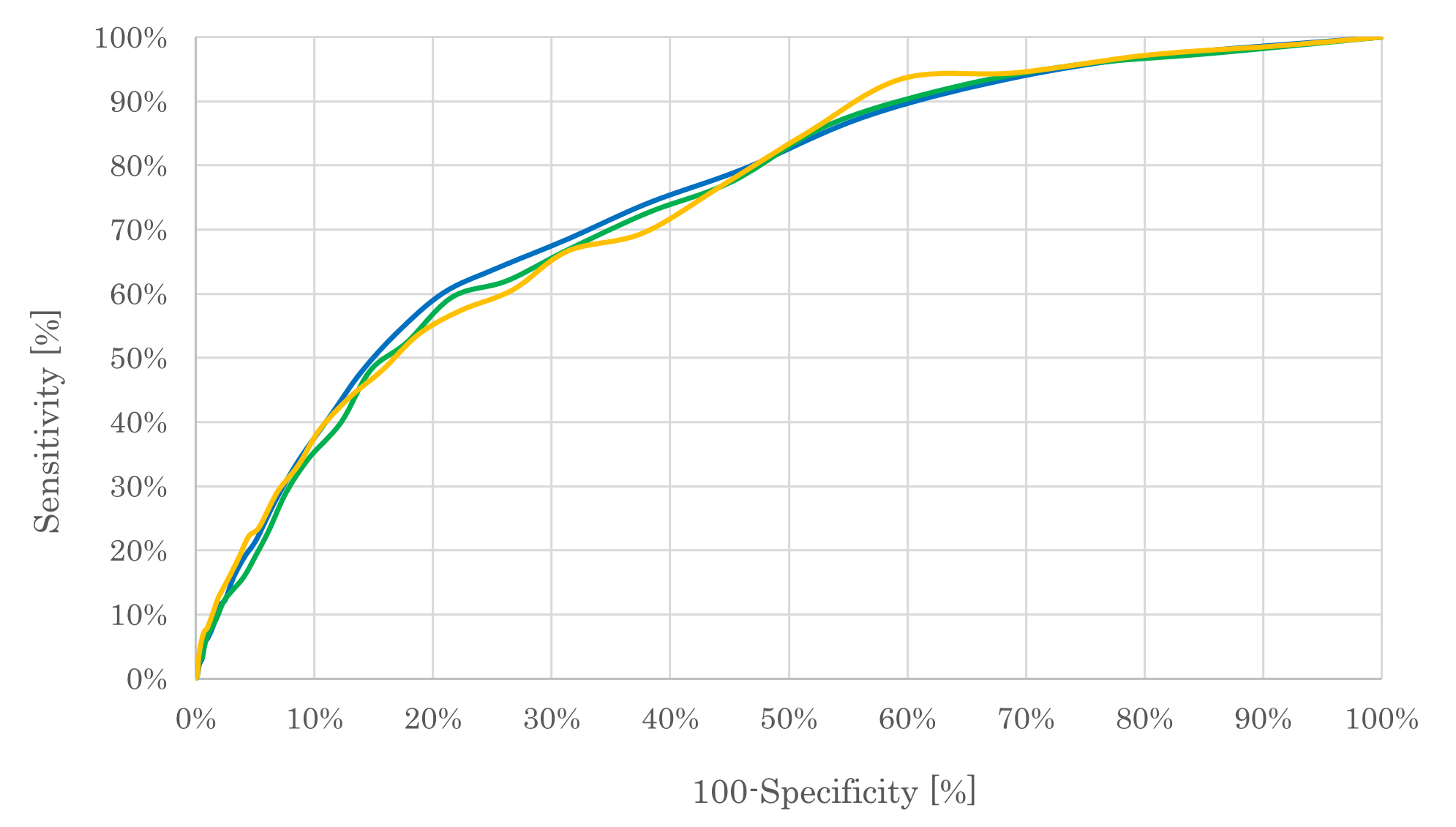

Supplement: S6 Fig — (TIFF) [file pone.0297361.s006.tiff]

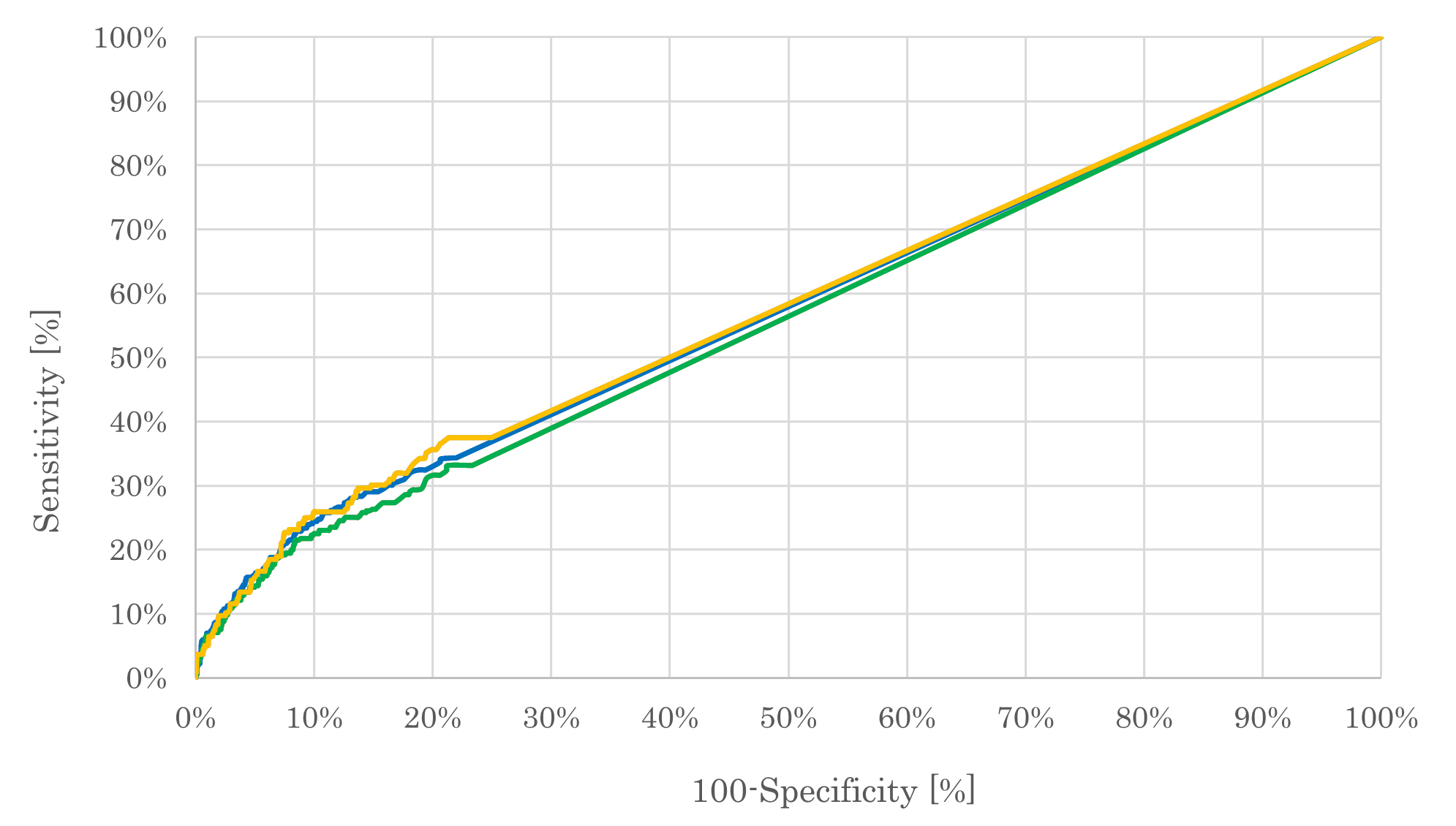

Supplement: S7 Fig — (TIFF) [file pone.0297361.s007.tiff]

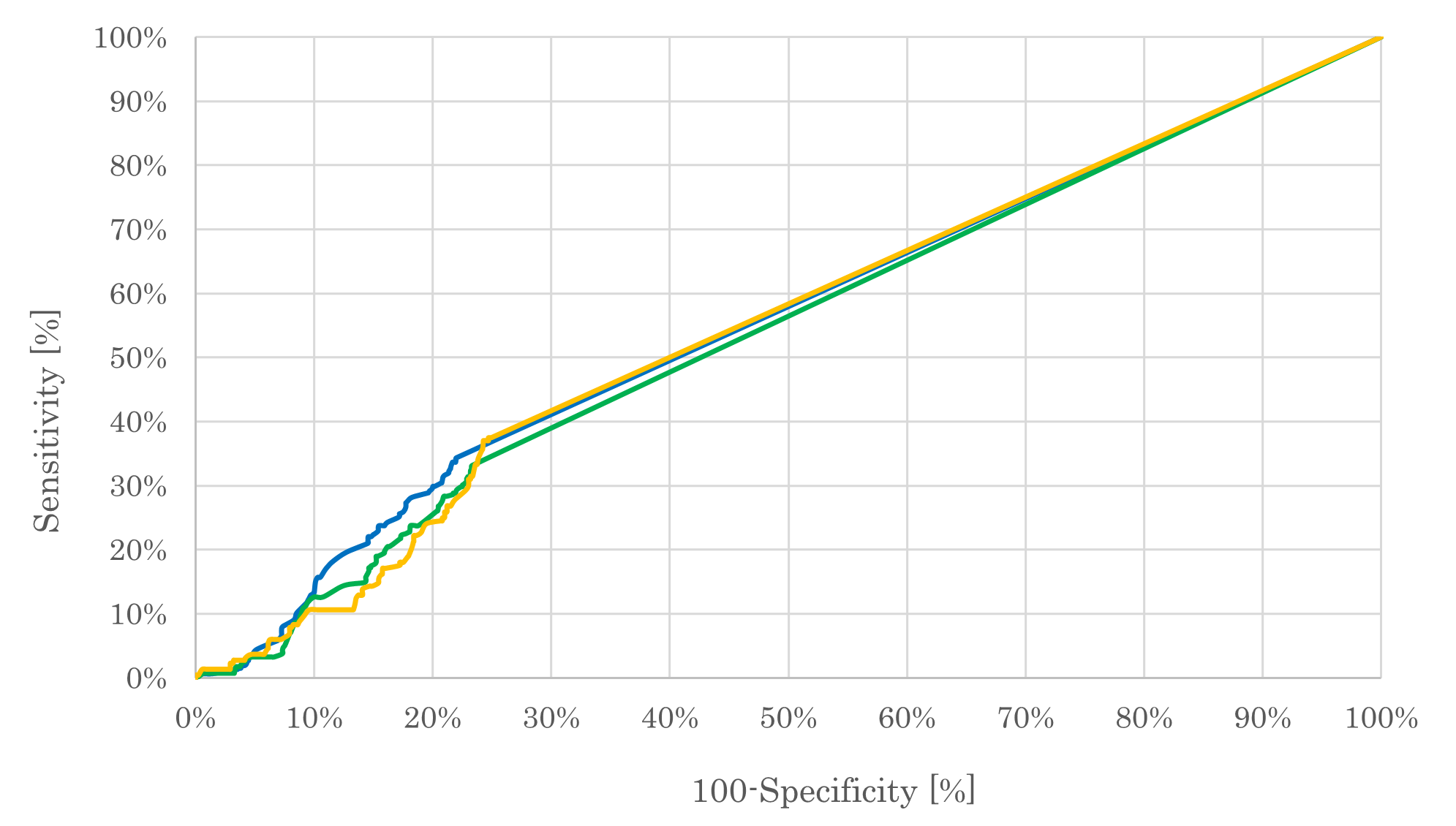

Supplement: S8 Fig — (TIFF) [file pone.0297361.s008.tiff]
