# Supplementary material for: Detecting possible pairs of materials for composites using a material word co-occurrence network
Source: PLoS One. 2024 Jan 26;19(1):e0297361. doi: 10.1371/journal.pone.0297361 (PMC10817182; doi:10.1371/journal.pone.0297361)
Supplement: S1 Table — (DOCX) [file pone.0297361.s009.docx]

**Table S1.** 100 material words and the number of papers in which they were found.

| **Material word(s)** | **The number of papers containing the word(s)** | **Material word(s)** | **The number of papers containing the word(s)** | **Material word(s)** | **The number of papers containing the word(s)** | **Material keyword(s)** | **The number of papers containing the word(s)** |
| --- | --- | --- | --- | --- | --- | --- | --- |
| graphene | 8736 | Fe_3_O_4_ | 971 | polyimide | 436 | glucose oxidase | 124 |
| epoxy | 7980 | polyurethane | 927 | polyamide | 423 | cyclodextrin | 121 |
| carbon nanotube | 6386 | polyvinyl alcohol (PVA) | 881 | palladium | 417 | polydopamine | 115 |
| silica (SiO_2_) | 5741 | tin oxide (SnO_2_) | 836 | chromium | 391 | lithium chloride (LiCl) | 114 |
| titanium dioxide (TiO_2_) | 4382 | manganese oxide (MnO_2_) | 802 | Co_3_O_4_ | 368 | polyvinylidene | 103 |
| aluminum | 3500 | carbonitride (C_3_N_4_) | 763 | zirconium | 361 | silver nitrate (AgNO_3_) | 95 |
| cellulose | 3359 | polypyrrole | 722 | platinum | 334 | pyridine | 91 |
| graphite | 3269 | molybdenum disulfide (MoS_2_) | 709 | chitin | 327 | polysiloxane | 89 |
| polyaniline (PANi) | 2827 | silane | 709 | LiFePO_4_ | 314 | carboxymethyl cellulose | 85 |
| copper | 2475 | polystyrene | 707 | magnesium oxide (MgO) | 300 | polythiophene | 82 |
| Al_2_O_3_ | 1965 | boron carbide (B_4_C) | 691 | cobalt ferrite (CoFe_2_O_4_) | 289 | vanadium phosphate | 74 |
| polypropylene | 1952 | barium titanate (BaTiO_3_) | 671 | cerium oxide (CeO_2_) | 279 | polyacrylamide | 73 |
| polyethylene | 1863 | zirconium dioxide (ZrO_2_) | 643 | polylactic acid | 257 | polysulfone | 71 |
| silver | 1703 | polymethylmethacrylate (PMMA) | 640 | polysaccharide | 234 | kaolinite | 69 |
| polyester | 1643 | calcium phosphate | 629 | fullerene | 223 | silsesquioxane | 67 |
| zinc oxide (ZnO) | 1637 | titanium diboride (TiB_2_) | 542 | polyelectrolyte | 219 | vinylpyridine | 65 |
| alumina | 1527 | Fe_2_O_3_ | 541 | polycaprolactone | 205 | polyolefin | 60 |
| nickel | 1428 | polyvinylidene fluoride (PVDF) | 534 | polycarbonate | 201 | calcium silicate | 58 |
| aluminum | 1401 | calcium carbonate (CaCO_3_) | 521 | polylactide | 196 | nickel hydroxide | 57 |
| hydrogel | 1396 | copper oxide (CuO) | 473 | vanadium oxide (V_2_O_5_) | 189 | polybutadiene | 57 |
| diamond | 1299 | nickel oxide (NiO) | 462 | calcium chloride (CaCl_2_) | 159 | polyetherimide | 53 |
| chitosan | 1295 | polyvinyl chloride (PVC) | 454 | molybdenum trioxide (MoO_3_) | 159 | polyvinylpyrrolidone | 51 |
| gold | 1221 | nylon | 453 | polyacrylonitrile | 153 | cadmium sulfide | 50 |
| collagen | 1190 | Li_3_V_2_ | 449 | melamine | 143 | FeCl_3_ | 46 |
| zeolite | 1115 | boron nitride | 439 | polydimethylsiloxane | 133 | calcium hydroxide | 41 |
