# Supplementary material for: Detecting possible pairs of materials for composites using a material word co-occurrence network
Source: PLoS One. 2024 Jan 26;19(1):e0297361. doi: 10.1371/journal.pone.0297361 (PMC10817182; doi:10.1371/journal.pone.0297361)
Supplement: S2 Table — (DOCX) [file pone.0297361.s010.docx]

**Table S2.** The number of co-occurring material word(s) and the percentage of co-occurrence of 100 material word(s).

| **Material word(s)** | **The number of co-occurring material words** | | **The percentage of co-occurrence** | **Material word(s)** | **The number of co-occurring material words** | | **The percentage of co-occurrence** |
| --- | --- | --- | --- | --- | --- | --- | --- |
|  | **2012** | **2015** |  |  | **2012** | **2015** |  |
| silica (SiO_2_) | 78 | 85 | 33.3% | polyacrylonitrile | 21 | 26 | 6.4% |
| carbon nanotube | 76 | 82 | 26.1% | silver nitrate (AgNO_3_) | 20 | 28 | 10.1% |
| graphite | 64 | 73 | 25.7% | polysiloxane | 20 | 23 | 3.8% |
| polyethylene | 63 | 74 | 30.6% | polyvinylidene | 20 | 22 | 2.5% |
| titanium dioxide (TiO_2_) | 63 | 70 | 19.4% | nickel oxide (NiO) | 19 | 26 | 8.8% |
| epoxy | 62 | 72 | 27.0% | tin oxide (SnO_2_) | 19 | 25 | 7.5% |
| silane | 58 | 64 | 14.6% | cerium oxide (CeO_2_) | 19 | 24 | 6.3% |
| polystyrene | 57 | 66 | 21.4% | polyvinyl chloride (PVC) | 18 | 25 | 8.6% |
| pluminum | 57 | 62 | 11.9% | FeCl_3_ | 18 | 21 | 3.7% |
| polyaniline (PANi) | 54 | 65 | 24.4% | magnesium oxide (MgO) | 17 | 24 | 8.5% |
| copper | 52 | 67 | 31.9% | polyvinylidene fluoride (PVDF) | 17 | 23 | 7.3% |
| silver | 50 | 64 | 28.6% | polysulfone | 16 | 23 | 8.4% |
| alumina | 50 | 62 | 24.5% | polyvinylpyrrolidone | 16 | 19 | 3.6% |
| cellulose | 50 | 60 | 20.4% | polycaprolactone | 16 | 18 | 2.4% |
| nickel | 49 | 57 | 16.0% | boron nitride | 15 | 23 | 9.5% |
| aluminum oxide (Al_2_O_3_) | 47 | 55 | 15.4% | polybutadiene | 15 | 17 | 2.4% |
| graphene | 43 | 80 | 66.1% | silsesquioxane | 14 | 24 | 11.8% |
| gold | 42 | 48 | 10.5% | polylactic acid | 14 | 21 | 8.2% |
| polyester | 40 | 46 | 10.2% | polysaccharide | 14 | 21 | 8.2% |
| polyvinyl alcohol (PVA) | 40 | 45 | 8.5% | polydimethylsiloxane | 14 | 19 | 5.9% |
| polyurethane | 40 | 44 | 6.8% | chitin | 13 | 18 | 5.8% |
| polypropylene | 39 | 45 | 10.0% | boron carbide (B_4_C) | 13 | 16 | 3.5% |
| aluminum | 39 | 43 | 6.7% | titanium diboride (TiB_2_) | 13 | 15 | 2.3% |
| polypyrrole | 36 | 42 | 9.5% | fullerene | 12 | 18 | 6.9% |
| zinc oxide (ZnO) | 34 | 44 | 15.4% | cobalt ferrite (CoFe_2_O_4_) | 12 | 18 | 6.9% |
| calcium phosphate | 34 | 39 | 7.7% | polythiophene | 11 | 17 | 6.8% |
| diamond | 31 | 36 | 7.4% | cyclodextrin | 11 | 13 | 2.3% |
| chitosan | 30 | 46 | 23.2% | molybdenum disulfide (MoS_2_) | 10 | 23 | 14.6% |
| polymethylmethacrylate (PMMA) | 30 | 36 | 8.7% | LiFePO_4_ | 10 | 17 | 7.9% |
| barium titanate (BaTiO_3_) | 29 | 34 | 7.1% | polyacrylamide | 10 | 16 | 6.7% |
| nylon | 28 | 31 | 4.2% | kaolinite | 10 | 14 | 4.5% |
| zirconium | 27 | 37 | 13.9% | polyolefin | 10 | 14 | 4.5% |
| polycarbonate | 27 | 30 | 4.2% | Co_3_O_4_ | 9 | 20 | 12.2% |
| zeolite | 26 | 34 | 11.0% | vanadium oxide (V_2_O_5_) | 9 | 14 | 5.6% |
| platinum | 26 | 33 | 9.6% | vinylpyridine | 9 | 9 | 0.0% |
| zirconium dioxide (ZrO_2_) | 26 | 32 | 8.2% | melamine | 8 | 12 | 4.4% |
| polyamide | 26 | 31 | 6.8% | carboxymethyl cellulose | 7 | 18 | 12.0% |
| polyimide | 26 | 31 | 6.8% | polylactide | 7 | 14 | 7.6% |
| hydrogel | 25 | 35 | 13.5% | Li_3_V_2_ | 7 | 13 | 6.5% |
| palladium | 24 | 33 | 12.0% | calcium chloride (CaCl_2_) | 7 | 11 | 4.3% |
| collagen | 24 | 30 | 8.0% | polyetherimide | 7 | 10 | 3.3% |
| polyelectrolyte | 24 | 26 | 2.7% | molybdenum trioxide (MoO_3_) | 5 | 13 | 8.5% |
| Fe_3_O_4_ | 23 | 39 | 21.1% | lithium chloride (LiCl) | 5 | 9 | 4.3% |
| pyridine | 23 | 27 | 5.3% | carbon nitride (C_3_N_4_) | 4 | 13 | 9.5% |
| chromium | 23 | 26 | 3.9% | cadmium sulfide | 4 | 7 | 3.2% |
| calcium carbonate (CaCO_3_) | 22 | 26 | 5.2% | nickel hydroxide | 4 | 4 | 0.0% |
| copper oxide (CuO) | 21 | 34 | 16.7% | polydopamine | 3 | 15 | 12.5% |
| Fe_2_O_3_ | 21 | 28 | 9.0% | calcium hydroxide | 3 | 4 | 1.0% |
| glucose oxidase | 21 | 27 | 7.7% | calcium silicate | 1 | 3 | 2.0% |
| manganese oxide (MnO_2_) | 21 | 26 | 6.4% | vanadium phosphate | 1 | 2 | 1.0% |
