# Supplementary material for: Detecting possible pairs of materials for composites using a material word co-occurrence network
Source: PLoS One. 2024 Jan 26;19(1):e0297361. doi: 10.1371/journal.pone.0297361 (PMC10817182; doi:10.1371/journal.pone.0297361)
Supplement: S3 Table — (DOCX) [file pone.0297361.s011.docx]

**Table S3.** The number of scientific papers in which each of the 100 material words occurred.

| **Network index** | Training period | **Cutoff**  **(Condition of positive)** | **AUC** | **Average accuracy** |
| --- | --- | --- | --- | --- |
| CN | TRP1 | CN≧7 | 75.5% | 65.4% |
|  | TRP2 | CN≧9 | 77.9% | 68.5% |
|  | TRP3 | CN≧5 | 77.7% | 68.9% |
| JC | TRP1 | JC≧0.091 | 70.5% | 62.7% |
|  | TRP2 | JC≧0.200 | 70.4% | 66.2% |
|  | TRP3 | JC≧0.186 | 71.3% | 66.6% |
| RA | TRP1 | RA≧0.055 | 74.0% | 66.2% |
|  | TRP2 | RA≧0.130 | 74.9% | 68.1% |
|  | TRP3 | RA≧0.076 | 75.6% | 69.0% |
| AA | TRP1 | AA≧1.048 | 73.3% | 66.0% |
|  | TRP2 | AA≧2.195 | 74.9% | 68.6% |
|  | TRP3 | AA≧1.207 | 73.4% | 68.9% |
| PA | TRP1 | PA≧348 | 72.4% | 67.2% |
|  | TRP2 | PA≧500 | 74.0% | 68.9% |
|  | TRP3 | PA≧368 | 69.0% | 63.2% |
| CNSH | TRP1 | CNSH≧8 | 77.9% | 69.7% |
|  | TRP2 | CNSH≧9 | 76.9% | 69.0% |
|  | TRP3 | CNSH≧8 | 77.0% | 67.7% |
| RASH | TRP1 | RASH≧0.064 | 57.5% | 57.5% |
|  | TRP2 | RASH≧0.083 | 56.2% | 56.6% |
|  | TRP3 | RASH≧0.020 | 58.0% | 58.1% |
| WIC | TRP1 | WIC≧0.014 | 55.8% | 56.2% |
|  | TRP2 | WIC≧0.071 | 54.2% | 54.9% |
|  | TRP3 | WIC≧0.111 | 54.9% | 56.3% |
