# Supplementary material for: Detecting possible pairs of materials for composites using a material word co-occurrence network
Source: PLoS One. 2024 Jan 26;19(1):e0297361. doi: 10.1371/journal.pone.0297361 (PMC10817182; doi:10.1371/journal.pone.0297361)
Supplement: S4 Table — (DOCX) [file pone.0297361.s012.docx]

**Table S4.** The number of scientific papers in which each of the 100 material words occurred.

| **Material word(s)** | **The number of co-occurring material words in 2020/8/15** | **The percentage of co-occurrence from 2016/1/1 to 2020/8/15** | **Material word(s)** | **The number of co-occurring material words in 2020/8/15** | **The percentage of co-occurrence from 2016/1/1 to 2020/8/15** |
| --- | --- | --- | --- | --- | --- |
| graphene | 93 | 68.4% | polyimide | 41 | 14.7% |
| epoxy | 77 | 18.5% | polyamide | 42 | 16.2% |
| carbon nanotube | 91 | 52.9% | palladium | 35 | 3.0% |
| silica (SiO_2_) | 88 | 21.4% | chromium | 36 | 13.7% |
| titanium dioxide (TiO_2_) | 79 | 31.0% | Co_3_O_4_ | 26 | 7.6% |
| aluminum | 74 | 32.4% | zirconium | 46 | 14.5% |
| cellulose | 76 | 41.0% | platinum | 42 | 13.6% |
| graphite | 81 | 30.8% | chitin | 22 | 4.9% |
| polyaniline (PANi) | 76 | 32.4% | LiFePO_4_ | 22 | 6.1% |
| copper | 79 | 37.5% | magnesium oxide (MgO) | 31 | 9.3% |
| Al_2_O_3_ | 62 | 15.9% | cobalt ferrite (CoFe_2_O_4_) | 25 | 8.6% |
| polypropylene | 51 | 11.1% | cerium oxide (CeO_2_) | 29 | 6.7% |
| polyethylene | 80 | 24.0% | polylactic acid | 35 | 17.9% |
| silver | 74 | 28.6% | polysaccharide | 28 | 9.0% |
| polyester | 51 | 9.4% | fullerene | 24 | 7.4% |
| zinc oxide (ZnO) | 62 | 32.7% | polyelectrolyte | 33 | 9.6% |
| alumina | 68 | 16.2% | polycaprolactone | 26 | 9.9% |
| nickel | 68 | 26.2% | polycarbonate | 37 | 10.1% |
| aluminum | 49 | 10.7% | polylactide | 18 | 4.7% |
| hydrogel | 46 | 17.2% | vanadium oxide (V_2_O_5_) | 16 | 2.4% |
| diamond | 43 | 11.1% | calcium chloride (CaCl_2_) | 14 | 3.4% |
| chitosan | 58 | 22.6% | molybdenum trioxide (MoO_3_) | 19 | 7.0% |
| gold | 55 | 13.7% | polyacrylonitrile | 31 | 6.8% |
| collagen | 38 | 11.6% | melamine | 26 | 16.1% |
| zeolite | 39 | 7.7% | polydimethylsiloxane | 30 | 13.8% |
| Fe_3_O_4_ | 54 | 25.0% | glucose oxidase | 29 | 2.8% |
| polyurethane | 50 | 10.9% | cyclodextrin | 17 | 4.7% |
| polyvinyl alcohol (PVA) | 53 | 14.8% | polydopamine | 37 | 26.2% |
| tin oxide (SnO_2_) | 79 | 31.0% | lithium chloride (LiCl) | 13 | 4.4% |
| manganese oxide (MnO_2_) | 32 | 8.2% | polyvinylidene | 67 | 39.6% |
| carbonitride (C_3_N_4_) | 32 | 22.1% | silver nitrate (AgNO_3_) | 34 | 8.5% |
| polypyrrole | 54 | 21.1% | pyridine | 46 | 14.5% |
| molybdenum disulfide (MoS_2_) | 38 | 19.7% | polysiloxane | 24 | 1.3% |
| silane | 71 | 20.0% | carboxymethyl cellulose | 29 | 13.6% |
| polystyrene | 72 | 18.2% | polythiophene | 19 | 2.4% |
| boron carbide (B_4_C) | 23 | 8.4% | vanadium phosphate | 2 | 0.0% |
| barium titanate (BaTiO_3_) | 36 | 3.1% | polyacrylamide | 24 | 9.6% |
| zirconium dioxide (ZrO_2_) | 42 | 14.9% | polysulfone | 24 | 1.3% |
| polymethylmethacrylate (PMMA) | 43 | 11.1% | kaolinite | 24 | 11.8% |
| calcium phosphate | 44 | 8.3% | silsesquioxane | 28 | 5.3% |
| titanium diboride (TiB_2_) | 17 | 2.4% | vinylpyridine | 14 | 5.6% |
| Fe_2_O_3_ | 43 | 21.1% | polyolefin | 17 | 3.5% |
| polyvinylidene fluoride (PVDF) | 35 | 15.8% | calcium silicate | 12 | 9.4% |
| calcium carbonate (CaCO_3_) | 41 | 20.5% | nickel hydroxide | 6 | 2.1% |
| copper oxide (CuO) | 43 | 13.8% | polybutadiene | 18 | 1.2% |
| nickel oxide (NiO) | 28 | 2.7% | polyetherimide | 10 | 0.0% |
| polyvinyl chloride (PVC) | 36 | 14.9% | polyvinylpyrrolidone | 26 | 8.8% |
| nylon | 33 | 2.9% | cadmium sulfide | 13 | 6.5% |
| Li_3_V_2_ | 15 | 2.3% | FeCl_3_ | 29 | 10.3% |
| boron nitride | 39 | 21.1% | calcium hydroxide | 6 | 2.1% |
